# Anthelmintic resistance in gastrointestinal nematodes on communally reared sheep farms of the King Sabata Dalindyebo Municipality, South Africa

**DOI:** 10.1007/s00436-025-08532-x

**Published:** 2025-08-05

**Authors:** Songezo Mavundela, William Diymba Dzemo, Oriel Thekisoe

**Affiliations:** 1 Departmanent of Biological and Environmental Sciences, Walter Sisulu University, Private Bag X1, Mthatha, 5117 South Africa; 2 Unit for Environmental Sciences and Management, North-West University, Potchefstroom, 2531 South Africa

**Keywords:** Benzimidazole, Helminth, Small ruminants, Macrocyclic lactone, Imidazothiazole, FECRT

## Abstract

Anthelmintic drugs are readily accessible to farmers and have been widely used to control gastrointestinal nematode (GIN) infections in communally reared sheep in the Eastern Cape Province (ECP), South Africa. Indiscriminate and prolonged use of these drugs has inevitably contributed to the selection of resistant GIN populations. This study aimed to assess the anthelmintic resistance status of GINs in communally reared sheep farms in King Sabata Dalindyebo Local Municipality, ECP. A semi-structured questionnaire was used to collect data on farm characteristics and helminth control practices. Faecal egg count reduction tests were performed on 259 lambs selected from eight communal farms. Lambs with egg per gram count > 150 were treated with anthelmintics (co-formulation of albendazole + closantel, and levamisole) currently used on the farms as well as an alternative anthelmintic from a different drug class (ivermectin, and a co-formulation of levamisole + praziquantel). Several suboptimal helminth control practices were identified that may have compromised the accurate administration of anthelmintics and effective management of deworming. These include the advanced age (> 65 years) of farmers: failure to weigh sheep prior to drug administration, use of syringes (5 ml/10 ml) instead of drench guns, and absence of a drenching and shifting approach. Helminths eggs from the genera *Moniezia*, *Strongyloides*, *Trichuris*, and *Haemonchus* were identified in both pre-and post-treatment faecal samples. Coprological culture of post-treatment samples, followed by morphological and molecular identification of larvae, confirmed the presence of *Haemonchus contortus.* This GIN species exhibited resistance to all the anthelmintic formulations assessed in this study. These findings highlight the urgent need for improved management strategies to mitigate resistance and promote sustainable worm control in communal sheep farming areas of ECP.

**Supplementary Information:**

The online version contains supplementary material available at 10.1007/s00436-025-08532-x.

## Introduction

South Africa (SA) is one of the largest sheep-producing countries globally, with the Eastern Cape Province (ECP), accounting for the majority (29%) of sheep production (Chagas et al. 2022; Moyo and Ravhuhali 2023). Sheep in this region are primarily raised in communal areas, where animals from different farmers graze together on unfenced land (Moyo and Ravhuhali 2023). Sheep production in communal areas of the ECP by resource-poor farmers faces numerous challenges, including feed shortages, poor infrastructure, limited veterinary support, inadequate grazing land, restricted market access, water scarcity, high treatment costs, livestock theft, diseases, and parasitism caused by helminth infections (Mthi and Nyangiwe 2018; Mphahlele et al. 2019; Emsley 2021; Morinket 2022). Heavy helminth infections in sheep significantly impact productivity, leading to weight loss, weakened immunity, reduced meat and wool quality, decreased fertility, anaemia, lower milk production, and increased morbidity and mortality rates (Mphahlele et al. 2019). Gastrointestinal nematodes from the family Trichostrongylidae are recognised as major contributors to disease complexes, production losses, and economic challenges in the SA sheep industry (Tsotetsi et al. 2013). The genus *Haemonchus*, within the family Trichostrongylidae, includes the most pathogenic nematodes of ruminants, with *H. contortus* being the most prominent species (Roeber et al. 2013; Jansen et al. 2022). *Haemonchus contortus*, a blood-sucking parasite of the abomasum, is the most clinically and economically significant gastrointestinal nematode (GIN) species affecting small ruminants (Chagas et al. 2022). In SA, haemonchosis has been linked to sheep losses estimated at approximately USD 29 million, causing significant declines in small ruminant production (Vatta et al. 2001; Van Wyk & Bath 2002).

Worldwide, the primary strategy for controlling GINs in small ruminants is the use of anthelmintic drugs (FAO 2004; Muchiut et al. 2018; Verma et al. 2018; George et al. 2021; Mphahlele et al. 2021; Chagas et al. 2022). In SA, the most commonly used formulations for managing helminth infections are albendazole, ivermectin, and levamisole (Tsotetsi et al. 2013; Bakunzi et al. 2013; Mphahlele et al. 2021; Emsley et al. 2023). Additionally, co-formulations of different anthelmintic drug classes, have also been employed (Vander Merwe 2021). Helminth infections and their control using anthelmintic drugs have significant economic implications for the sheep production industry (Tsotetsi et al. 2013; Kalacho and Kunta 2024). In Africa, annual treatment costs for helminth infections in sheep have been estimated at USD 77.9 million in Nigeria (Odeniran et al. 2021), USD 26 million in Kenya (Kagira and Kanyari 2001), and USD 45 million in SA (Chagas et al. 2022).

Despite the widespread use anthelmintics, GINs continue to pose a significant challenge in sheep production (Emsley et al. 2023). This issue is largely attributed to farmer’s inadequate skills in administering drugs, prolonged reliance on them, and the use of poor-quality formulations (Besier and Love 2003; Tsotetsi et al. 2013; Mthi et al. 2021; Emsley et al. 2023). In SA, anthelmintics are readily accessible and are often administered by farmers without veterinary supervision (Larsen and Jansen 2012). Consequently, the repeated and indiscriminate use of these drugs has increased selection pressure for resistant worm strains, ultimately contributing to the development of anthelmintic resistance (AHR) (Mphahlele et al. 2019). Anthelmintic resistance is a genetically acquired reduction in the sensitivity of a parasite population to an anthelminthic, evidenced by repeated anthelmintic control failures at the manufacturer’s recommended concentration (FAO 2004; Mphahlele et al. 2019; Dzemo et al. 2022). This phenomenon has been documented in sheep across several countries worldwide, including Australia (Preston et al. 2019), the UK (Crilly et al. 2023), the USA (Voigt et al. 2022), Asia (Kour et al. 2025), Brazil (Bassetto et al. 2024), China (Hou et al. 2022), as well as in SA (Van Wyk et al. 1999; Bakunzi 2003; Tsotetsi et al. 2013; Mphahlele et al. 2021; Vander Merwe 2021). The widespread presence of AHR in GINs of sheep poses a significant threat to animal health and production worldwide (Fissiha and Kinde 2021). Moreover, the high costs and technical challenges associated with developing new anthelmintic drugs have made this issue a critical global concern (Fissiha and Kinde 2021; Dzemo et al. 2022). In several countries, including Australia, the UK, New Zealand, and SA, anthelmintic resistance has become so severe that it has led to the closure of sheep farms (Mphahlele et al. 2021). In SA, resistance to ivermectin, albendazole, and levamisole in *H. contortus* and *Trichostrongylus* spp. have been reported on small-scale farms in Gauteng Province (Tsotetsi et al. 2013). In the Free State Province, Vander Merwe (2021) identified *H. contortus* exhibiting varying levels of resistance to different anthelmintic classes. Mphahlele et al. (2021) reported that 90% of sheep on communal farms in Limpopo Province harboured nematode strains of *H. contortus* or *Trichostrongylus* spp. that were resistant to at least one anthelmintic class (levamisole, benzimidazoles, or macrocyclic lactones). Similarly, resistance to benzimidazoles, levamisole, and macrocyclic lactones in gastrointestinal nematodes of sheep and goats has been documented on small household farms in North-West Province (Emsley 2021).

Despite the ECP being the largest producer of communally reared sheep in SA (Moyo and Ravhuhali 2023), research on AHR in this region remains limited (Mthi and Nyangiwe 2018; Jansen et al. 2022). Regular assessment and monitoring of AHR are crucial for understanding the parasite burden within sheep flocks and supporting farmers in making informed decisions regarding anthelmintic use for effective worm control. Therefore, this study aimed to determine the AHR status in communally reared sheep in the King Sabata Dalindyebo (KSD) Local Municipality of the ECP, SA.

## Material and methods

### Study area and design

**Fig. 1:**
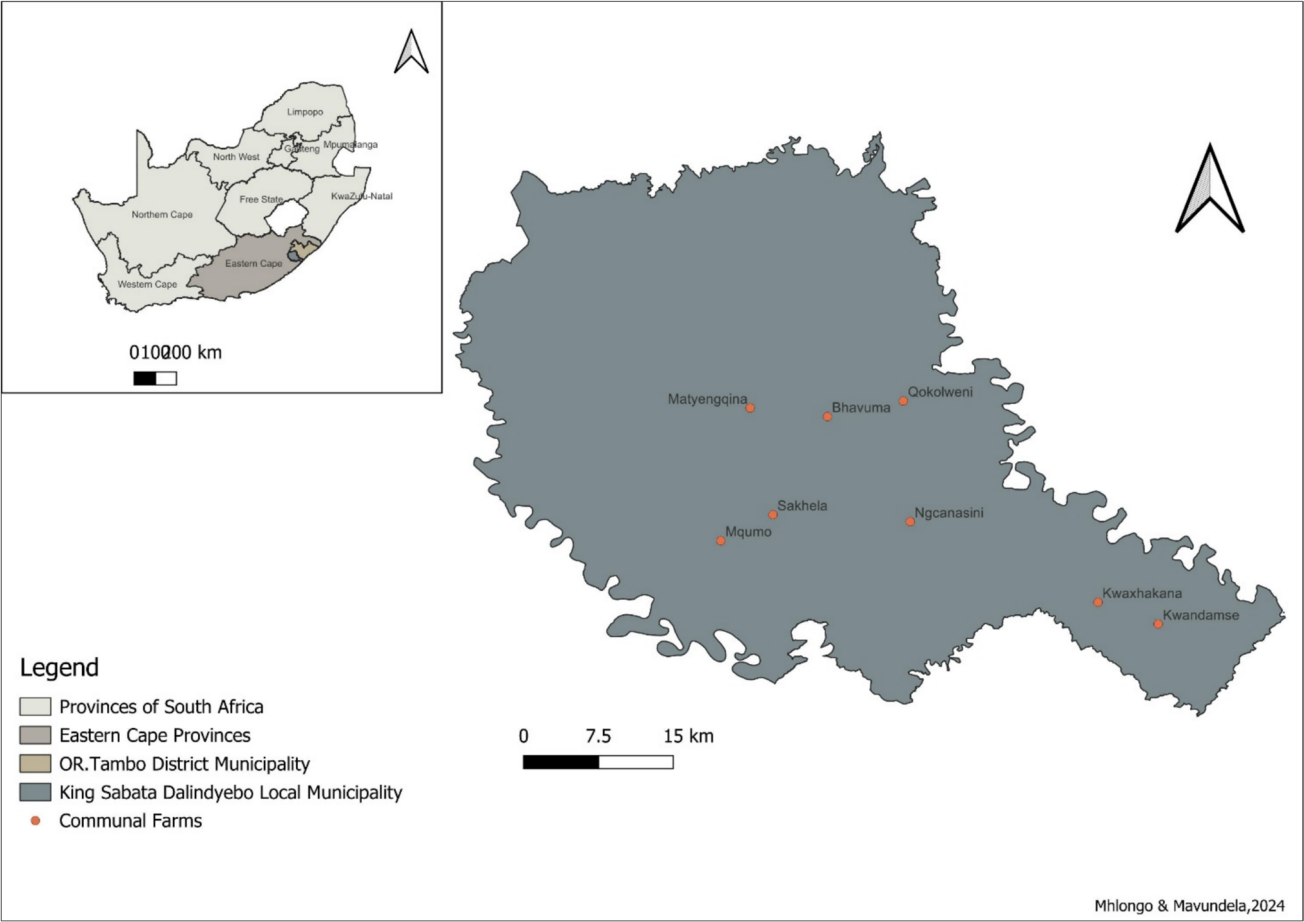
Map of the King Sabata Dalindyebo local municipality showing communal farms selected for assessing anthelminthic resistance development

The KSD local municipality in the ECP of SA (31° 44′ 54″ S 28° 44′ 40″ E) (Fig. 1), features a landscape of hills and mountains, with an average altitude of 764 m above sea level beyond the Indian Ocean coastline. Climatic conditions vary with distance and elevation from the Indian Ocean. Coastal areas experience a tropical climate, while inland areas have a temperate climate. Annual rainfall ranges from approximately 800–871 mm in summer (December to February) and 350 mm in winter (June to August) along the coast, decreasing further inland. The mean temperature is approximately 30 °C during the summer months and 16 °C during the winter months (Integrated Development Plan 2017). The vegetation is predominantly composed of shrublands (coastal), forests and grassland (inland), and diverse grass species, including *Digitaria eriantha*, *Paspalum*, *Cynodon dactylon*, *Eragrostis plana*, and *festuca rubra* (Nyangiwe et al. 2013), that are suitable for livestock rearing. Livestock (cattle, sheep, and goats) rearing is the main agricultural activity. The study area has an estimated sheep population of approximately 750,000 consisting mainly of Dohne-Merino and Doper-Merino sheep cross breeds (Cloete and Olivier 2010). Most of the livestock production is under the informal subsistence farming system in which sheep are kept mostly in communal areas. In such areas, farmers have no exclusive land tenure, and natural resources such as grazing land are shared and managed collectively (Dzemo et al. 2024). Other agricultural activities practised within the municipality include crop farming and forestry (O.R. Tambo Draft Business Plan 2015).

Prior to commencement of the study, an authorization letter and data, including contact details and location of communal sheep farmers, were obtained from the local District Veterinary Services office. Farmers who kept more than 100 animals and were exclusively using anthelmintic drugs for the control of worms were purposively pre-selected for the study. A semi-structured questionnaire was administered to the selected farmers to gather data on sheep farm characteristics, worm control approaches, anthelmintic drug usage, and application practices.

### Faecal sample collection

Faecal samples were collected from eight selected sheep farms between March and August 2024 (day 0 pre-treatment, and day 14 post-treatment; during the second visit to the farms). On these farms, only lambs aged 6–12 months old were sampled. The sampled lambs had not received any anthelmintic treatment for at least 8 weeks before faecal collection (Coles et al. 2006). Faecal samples were collected rectally from a total of 259 lambs between 05:30 and 07:30 a.m., before they were released from the sheepfold to graze. During the faecal collection process, lambs were restrained in the sheepfold to stand upright. Faecal sample collection was performed according to Zajac and Conboy (2012). All faecal samples collected were placed into plastic sealed bags, labelled with the farm name, date of collection, and animal identification number, and stored in a cooler box with an ice pack. After collection, the faecal samples were immediately transported to the Zoology Laboratory of the Department of Biological and Environmental Sciences at the Walter Sisulu University, Mthatha campus, for coprological examination.

### Coprological examination

Upon arrival at the laboratory, the McMaster technique (Zajac and Conboy 2012) was used to determine the presence of helminth eggs in the collected faecal samples. A sodium chloride floatation solution was prepared according to Zajac and Conboy (2012). The faecal sample from each sealed plastic bag was kneaded to homogenise the pellets. Two grams of the faecal sample were weighed into a 100-ml plastic beaker, using a digital laboratory scale (KERN ABJ-NM/ABS-N™). Approximately 28 ml of the sodium chloride floatation solution was added to the 2 g of faecal sample and soaked for 5–10 min until the faeces soften. The sample was stirred and agitated to break pellets into smaller pieces, separating eggs and oocysts from faecal debris. The homogenised mixture was poured through a 200-µm tea sieve into a clean, labelled 100-ml plastic beaker. Aliquots were then transferred into two McMaster chambers using a Pasteur pipette, and the loaded chambers were left undisturbed for about 5 min to allow eggs to float (Zajac and Conboy 2012). The eggs that encountered the upper glass of the chambers were visualised under a compound microscope (Zeiss® Upright Microscope Primo Star, Oberkochen, Germany). Using a magnification of × 100, all the visible eggs were counted and reported as eggs per gram (EPG) of faeces by multiplying the number of eggs by a factor of 50. Each egg observed and counted represented 50 EPG of faeces (Ministry of Agriculture Fisheries and Food, 1986; Zajac and Conboy 2012). Morphological identification of the eggs was performed as described in Zajac and Conboy (2012).

### Assessing for anthelmintic resistance

All 259 lambs sampled from the eight farms had a faecal egg count burden ≥ 150 EPG and were eligible for the assessment of AHR development (FAO 2004; Mphahlele et al. 2021). The weights of the lambs were estimated using a heart girth method (Pater 2007), as follows:

$$\mathrm{Weight}\;(\mathrm{lbs})\;=\frac{\mathrm{Heart}\;\mathrm{girth}\;(\mathrm{inches})\;\times\:\mathrm{Heart}\;\mathrm{girth}\;(\mathrm{inches})\;\times\;\mathrm{Body}\;\mathrm{length}\;(\mathrm{inches})}{300}$$

The resulting weights in pounds were converted to kilograms by multiplying with a factor of 0.45 (Pater 2007). A drench gun was then calibrated to ensure the lambs received the correct dose of the anthelminthic drug. The calibration was performed following the method described by Abbott et al. (2004), which recommends using a graduated measuring device and the actual anthelmintic drug to be administered rather than water. Furthermore, the Faffa Malan Chart (FAMACHA ^©^) eye-colour score was used to assess the level of anaemia in the lambs. Lambs were scored on a scale of 1–5 as follows: 1 = non-anaemic (very red), 2 = non-anaemic (slightly red), 3 = mildly anaemic (pale red), 4 = anaemic (pale), and 5 = severely anaemic (very pale) (Şahin et al. 2021). The presence of anaemia is associated with heavy worm burden in sheep (Şahin et al. 2021). A total of 182 lambs (EPG ≥ 150 and FAMACHA^©^ score ≥ 3), were assessed for anthelmintic drug resistance using World Association for the Advancement of Veterinary Parasitology (WAAVP) guidelines by Kaplan et al. (2023). Lambs from each farm were ear-tagged and randomly assigned to one of two treatment groups: one receiving the anthelmintic drug currently used on the farm, another receiving an alternative anthelmintic with a different mode of action (Table 1). Each treatment group consisted of 10–15 lambs (FAO 2004; Kaplan et al. 2023). The anthelmintic drugs were purchased from registered local veterinary drug shops. These drugs are screened for quality, including the concentration of active ingredients, by the Department of Agriculture, Forestry, and Fisheries under the Agricultural Remedies Registrar Act 36 of 1947 (Table 1). Only batches that meet label claims are registered and made available for use in helminth control.

On day 14 post-treatment, faecal samples were collected, and a coprological examination was conducted to determine the EPG after treatment (FAO 2004; Zajac and Conboy 2012).

**Table 1:** Anthelmintic drugs administered orally to lambs sampled on communal farms in the King Sabata Dalidyebo local municipality

Farm name	Anthelmintic drug currently used on the farm		Alternative anthelmintic drug administered	
	Trade name (Dose; Reg. No. Act 36 of 1947)	Active ingredient(s) (Anthelmintic class)	Trade name (Dose; Reg. No. Act 36 of 1947)	Active ingredient(s) (Anthelmintic class)
Bhavuma	Prodose® Orange (2 ml/10 kg; G2101)	Albendazole 1.9% m/v + Closantel sodium 3.94% m/v (BZ + SLD)	Endo + Lint® (2 ml/10 kg; G3570)	Levamisole HCI 3.75% m/v + Praziquantel 1.88% m/v (IMD + ISN)
Matyengqina	Prodose® Orange (2 ml/10 kg; G2101)	Albendazole 1.9% m/v and Closantel sodium 3% m/v (BZ + SLD)	Ecomectin® (2.5 ml/10 kg; G2630)	Ivermectin 0.08% m/v (ML)
Mgqumo	Prodose® Orange (2 ml/10 kg; G2101)	Albendazole 1.9% m/v + Closantel sodium 3.94% m/v (BZ + SLD)	Ecomectin® (2.5 ml/10 kg; G2630)	Ivermectin 0.08% m/v (ML)
Sakhela	Prodose® Orange (2 ml/10 kg; G2101)	Albendazole 1.9% m/v + Closantel sodium 3.94% m/v (BZ + SLD)	Ecomectin® (2.5 ml/10 kg; G2630)	Ivermectin 0.08% m/v (ML)
Ngcanasini	Prodose® Red (3 ml/10 kg; G2102)	Levamisole hydrochloride 2.56% m/v (IMD)	Ecomectin® (2.5 ml/10 kg; G2630)	Ivermectin 0.08% m/v (ML)
Qokolweni	Prodose® Red (3 ml/10 kg; G2102)	Levamisole hydrochloride 2.56% m/v (IMD)	Ecomectin® (2.5 ml/10 kg; G2630)	Ivermectin 0.08% m/v (ML)
KwaXhakana	Prodose® Orange (2 ml/10 kg; G2101)	Albendazole 1.9% m/v + Closantel sodium 3.94% m/v (BZ + SLD)	Prodose® Red (3 ml/10 kg; G2102)	Levamisole hydrochloride 2.56% m/v (IMD)
Ngcanasini	Prodose® Orange (2 ml/10 kg; G2101)	Albendazole 1.9% m/v + Closantel sodium 3.94% m/v (BZ + SLD)	Ecomectin® (2.5 ml/10 kg; G2630)	Ivermectin 0.08% m/v (ML)

*BZ* + *SLD*, co-formulation of benzimidazoles and salicylanilides; *ISN* + *IMD*, co-formulation of imidazothiazoles + isoquinoline; *SLD*, formulation of salicylanilides; *ML*, formulation of macrocyclic lactones

### Larval culture and identification

#### Larval culture

Pre- and post-treatment faecal samples with high egg counts (EPG ≥ 300) were processed for faecal cultures using the method described by Van Wyk and Mayhew (2013) to identify third-stage (L_3_) nematode genera.

#### Morphological identification

The L_3_ larvae from faecal cultures were extracted and identified following the method of Van Wyk and Mayhew (2013). Larvae were examined using a digital compound microscope (Olympus® BX43 Clinical Microscope, Olympus Corporation, Tokyo, Japan) at × 100 magnification. Micrographs were captured, and measurements were taken for total larval length, sheath tail, and larval filament. These measurements were compared with dichotomous keys used to identify the L_3_ larvae of common nematodes in small ruminants (Van Wyk and Mayhew 2013). The remaining distilled water containing the L_3_ was stored at − 20 °C.

#### Molecular identification

Deoxyribonucleic acid (DNA) was extracted from third-stage larvae (L_3_) stored at − 20 °C, using a Zymo® research kit (Catalog No. D6016, Zymo Research Corp., Version 2.2.1). Frozen samples were thawed, and 1000 µL of the sample was mixed with 750 µL of BashingBead™ Buffer in a lysis tube, then processed in a tissue lyser for 10 min. Homogenisation was performed using a Prism™ R centrifuge, with serial processing through the Zymo-Spin™ III-F in different collection tubes.

The concentration and quality of the extracted DNA samples were assessed using a NanoDrop One spectrophotometer (Thermo Fischer Scientific Inc., USA) and stored at 4 °C.

To enable the molecular identification of various nematode species, the Internal Transcribed Spacer 1 (ITS1) (Powers et al. 1997) region was amplified using polymerase chain reaction (PCR). The PCR was performed in a 25 μL of mixture containing 12.5 µL of One Taq PCR Master Mix (Applied Biosytems, USA), 2 μL of a primer mix (1 μL each of the forward [5′-CGTAACAAGGTAGCTGTAG3′] and reverse [5′-TCCTCCGCTAAATGATATG-3′] primers), 8.5 μL of nuclease-free water (NFW), and 2 μL of the template DNA.

The PCR thermal cycler (MiniAmp.™ Plus, Life Technologies, Singapore) was programmed for an initial denaturation at 94 °C for 30 s, followed by 35 cycles of amplification, including denaturation at 94 °C for 30 s, annealing at 48 °C for 45 s, and extension at 68 °C for 1 min. The process concluded with a final extension at 68 °C for 5 min, after which the samples were held at 4 °C.

The PCR products were analysed using gel electrophoresis to confirm amplification success and determine DNA fragment sizes. A 1% agarose gel was prepared by dissolving 1 g of LE agarose in 100 mL of TAE buffer, heating the mixture, and adding ethidium bromide. The gel was set, placed in an electrophoresis chamber, and submerged in TAE buffer. Approximately, 5 µL of a 100 base pair DNA ladder, a negative control, and PCR products were loaded into separate wells. Electrophoresis was run at 100 V for 30 min, and the gel was visualised under UV light. The resulting image was recorded, and all PCR-positive samples were sent for sequencing at Inqaba Biotech Industries (Pretoria, South Africa).

### Data analysis

Data from the questionnaire were summarised and entered into Microsoft® Excel® 365 for Windows. Counts and percentages were calculated for various factors that might be plausibly associated with the development of AHR, including the sex and age of farmers, farming experience, sheep breeds reared, anthelmintics used on the farm, and helminth control practices.

Anthelmintic resistance was assessed using the faecal egg count reduction test percentage (FECRT%) formula, as described by Kochapakdee et al. (1995): FECRT% = 100 × (1 − [T2/T1]), where T2 and T1 represent the arithmetic mean of the treated group’s EPG counts post-treatment and pre-treatment, respectively, from the same animals (paired). Furthermore, individual EPG counts from day 0 (pre-treatment) and day 14 (post-treatment) were uploaded to a web application (http://www.fecrt.com) that employs a hybrid Frequentist/Bayesian analysis approach. The 90% confidence intervals (CIs) of the FECRT% were calculated using the Delta method (Levecke et al. 2018), WAAVP method (Coles et al. 1992), and Beta Negative Binomial version B (BNB-B) method (Denwood et al. 2023), following the recently updated WAAVP guidelines (Kaplan et al. 2023). Anthelmintic efficacy was classified as susceptible when FECRT% ≥ 95% and the lower 90% CI ≥ 90%, while resistance was identified when FECRT% < 90% and the lower 90% CI ≤ 90% (Kaplan et al. 2023).

Nucleotide sequences were extracted and converted from AB1 to FASTA format using Molecular Evolutionary Genetics Analysis Version 11 (MEGA11) for genetic identification of the nematodes. Mixed bases were edited to their appropriate base pairs (Tamura et al. 2011). The resulting sequences were subjected to the nucleotide Basic Local Alignment Search Tool (BLASTn) to align against references in the GenBank of National Center for Biotechnology Information (NCBI) (https://ncbi.nlm.nih.gov/BLASTn, accessed on 29 January 2025) for high-similarity sequences confirming the identity of the nematodes.

## Results

All farmers from the selected farms were males, over 65 years old, and had more than 10 years of farming experience. They primarily raised Doper-Merino and Dohne Merino sheep breeds. Benzimidazoles and salicylanilides formulations were commonly used for the control of helminths on these farms, though few farmers supplemented these treatments with plant extracts. Several inappropriate anthelmintic control practices were identified, potentially contributing to the reported anthelmintic control failure on these farms: These include: inaccurate drug administration, as sheep were not weighed before dosing, and inappropriate equipment (5 mL/10 mL syringes) was used instead of a calibrated drench gun; indiscriminate anthelmintic use, with farmers relying on the same drug for over 2 years and purchasing anthelmintics from veterinary drug shop without a prescription from a local veterinarian; and lack of strategic deworming, as the drench and shift approach was not implemented. Between three to five of these high-risk practices were observed on the sampled farms.

All 259 sampled lambs tested positive for gastrointestinal helminth (nematodes and cestodes), with an average EPG, equal or greater than 150 (EPG ≥ 150) (Table 2). Helminth eggs from three distinct genera including *Moniezia*, *Strongyloides*, and *Trichuris*, as well as Strongyle eggs were identified in the pre-treatment faecal samples (Table 2, Supplementary Figure S1). Strongyle and *Strongyloides* spp. eggs were the most commonly detected.

**Table 2:** Prevalence (%) and mean EPG of gastrointestinal helminths observed in communal sheep (*n* = 259) of the King Sabata Dalindyebo Municipality

Helminths	Infected lambs	Prevalence %	Mean EPG ± SE
**Cestodes**			
* Moniezia*	62	24	210.00 ± 14.53
**Nematodes**			
Strongyle	259	100	854.28 ± 66.25
* Strongyloides*	119	46	281.00 ± 33.99
* Trichuris*	15	6	150.00 ± 21.08
**Co-infections**			
Strongyle + *Moniezia*	62	24	545.00 ± 96.74
Strongyle + *Trichuris*	15	6	640.00 ± 134.54
Strongyle + *Strongyloides*	119	46	954.57 ± 141.00

*EPG*, eggs per gram; *SE*, standard error

Prodose® Orange (albendazole + closantel, 2 ml/10 kg) and Prodose® Red (levamisole, 3 ml/10 kg) were the anthelmintic drugs currently used on 75% (6/8) and 25% (2/8) of the farms sampled, respectively. Anthelmintic resistance was detected on all farms (100%) based on the FECRT% formula of Kochakpadee et al. (1995) and the clinical protocol outlined in the WAAVP guidelines (Kaplan et al. 2023) (Table 3). Anthelmintic resistance was also detected on all farms (100%) following the administration of alternative anthelmintic drugs with different modes of action: Ecomectin® (Ivermectin, 2.5 ml/10 kg) on 6/8 farms, Prodose® Red (levamisole, 3 ml/10 kg) on 1/8 farm, and Endo + Lint® (co-formulation of levamisole + praziquantel, 2 ml/10 kg) on 1/8 farm. Overall, multi-drug resistance was observed in GINs of sheep on communal areas of the KSD to Prodose® Orange (BZ + SLD) and Ecometin (ML) on 4/8 farms; Prodose® Orange (BZ + SLD) and Prodose® Red (IMD) on 1/8 farms; Prodose® Orange (BZ + SLD) and Endo + Lint (IMD + ISN) on 1/8 farm; and Prodose® Red (IMD) and Ecometin® (ML) on 2/8 farms (Table 3).

**Table 3:** Anthelmintic resistance status on eight communal sheep farms located in the King Sabatha Dalindyebo municipality, South Africa

Location/area and farm name	Anthelmintic treatment and the number of sheep treated (*n*)	% FECR^3^	Delta	90% CI	WAAVP	90% CI	BNB-B	Conclusion
Bhavuma	^1^Prodose® Orange (BZ + SLD) (*n* = 10)	− 123	R	− 222.8 to − 38.5%	R	− 254.3 to − 39.7%	R	R
	^2^Endo + Lint® (IMD + ISN) (*n* = 10)	73	R	54.6–86.7%	R	46.8–86.0%	R	R
Matyengqina	^1^Prodose® Orange (BZ + SLD) (*n* = 10)	50	R	18.1–74.6%	R	5.1–73.4%	R	R
	^2^Ecomectin® (ML) (*n* = 10)	52	R	15.1–74.5%	R	1.1–73.2%	R	R
Mgqumo	^1^Prodose® Orange (BZ + SLD) (*n* = 15)	85	R	79.8–89.8%	R	78.7–89.7%	R	R
	^2^Ecomectin® (ML) (*n* = 10)	83	R	72.4–91.9%	R	67.7–91.4%	R	R
Sakhela	^1^Prodose® Orange (BZ + SLD) (*n* = 11)	68	R	56.5–83.3%	R	51.7–82.9%	R	R
	^2^Ecomectin® (ML) (*n* = 10)	68	R	55–78.5%	R	51.4–78.5%	R	R
Ngcanasini	^1^Prodose® Red (IDM) (*n* = 10)	77	R	66–85.4%	R	62.7–85.3%	R	R
	^2^Ecomectin® (ML) (*n* = 13)	58	R	39.3–70.9%	R	35.2–70.5%	R	R
Qokolweni	^1^Prodose® Red (IDM) (*n* = 12)	− 143	R	− 280.8 to − 32.3%	R	− 327.5 to − 38.1%	R	R
	^2^Ecomectin® (ML) (*n* = 10)	− 9	R	− 81.3–47.7%	R	− 112.8–44.6%	R	R
KwaXhakana	^1^Prodose® Orange (BZ + SLD) (*n* = 11)	21	R	2.8–50.2%	R	− 3.5–50.1%	R	R
	^2^Prodose® Red (IDM) (*n* = 11)	70	R	55.5–79.7%	R	51.8–79.5%	R	R
KwaNdamse	^1^Prodose® Orange (BZ + SLD) (*n* = 12)	43	R	− 2.5–76.1%	R	− 23.1–73.3%	R	R
	^2^Ecomectin® (ML) (*n* = 13)	58	R	19.6–76.2%	R	7.7–74.5%	R	R

*BZ* + *SLD*, co-formulation of benzimidazoles and salicylanilides; *ISN* + *IMD*, co-formulation of imidazothiazoles + isoquinoline; *SLD*, formulation of salicylanilides; *ML*, formulation of macrocyclic lactones; ^1^Anthelmintic currently used on the farm; ^2^Alternative anthelmintic administered; ^3^FECR by Kochapakdee et al. (1995). Calculations were done employing the Delta (Levecke et al. 2018), WAAVP (Coles et al. 1992), and the beta negative binomial version B (BNB-B) method (Denwood et al. 2023), according to the recently published WAAVP guidelines (Kaplan et al. 2023)

Morphological analysis of L_3_ nematode larvae from pre-and post-treatment faecal cultures confirmed *H. contortus* as the dominant anthelmintic-resistant GINs species on farms of the KSD municipality (Supplementary Figure S2) (Table 4). *Haemonchus contortus* is of medium size, with a total length of approximately 680–730 µm (Supplementary Figure S2) (Van Wyk and Mayhew 2013; Abuelwafa et al. 2016), a sheath tail extension (STE) of 65–82 µm (Van Wyk and Mayhew 2013), and a filament measuring 10–15% of the STE (Van Wyk and Mayhew 2013). To supplement morphological identification, BLASTn results from the ITS1 gene fragment sequences were compared with the top three matches from the NCBI GenBank database (Supplementary Figure S3). The ITS1 gene representative sequences of *H. contortus* from three sheep farms (Mgqumo, Ngcwanguba, and Kwa-Xhakana) had a 92.58 to 96.01% similarity with the Russia, India, China, Egypt, and Iraq *H. contortus* with accessions nos. JN590055.1, KJ938044.1, MK402086.1, OP984151.1, and ON004114.1, respectively (Supplementary Figure S3).

**Table 4:** Prevalence (%) of gastrointestinal nematodes identified in the screened faecal samples from the selected sheep farms

Farm name	No. of lambs treated with anthelmintic drugs	Prevalence (%)					
		Haemonchus*		Trichuris*		Strongyloides*	
		Pre	Post	Pre	Post	Pre	Post
Bhavuma	20	100	100	5	-	75	12
Matyengqina	20	100	100	15	5	50	15
Mgqumo	25	100	100	8	-	48	8
Sakhela	21	100	100	4	-	52	19
Ngcanasini	26	100	100	3	-	38	-
Qokolweni	22	100	100	13	5	50	9
KwaXhakana	23	100	100	4	-	43	-
KwaNdamse	25	100	100	4	-	32	-
Total	182	100	100	7	1	48	11

*Identification of the *Heamonchus contortus* larvae was done after coproculture of the faecal samples. *Trichuris* and *Strongyloides* were identified based on the morphology of their eggs

## Discussion

Gastrointestinal nematode infections are a major constraint to sheep health and productivity worldwide (Sheferaw et al. 2021). These infections are primarily caused by *Haemonchus contortus*,* Trichostrongylus colubriformis*, *Bunostomum trigonocephalum*, and *Oesophagostomum* spp. (Worku et al. 2017). *Haemonchus contortus* is considered the most pathogenic helminth species affecting sheep (Van Wyk et al. 1999; Besier and Love 2003). It has been identified as the predominant GIN in communally reared sheep within the KSD local municipality of the ECP of SA. This finding is consistent with Horak (2003), who reported *H. contortus* as the most abundant GIN species in Merino sheep breed of the ECP of SA. Furthermore, Jansen et al. (2020) observed that *H. contortus* was the sole GIN species identified in faecal cultures from communally reared sheep in the ECP. The region’s favourable climatic conditions, characterised by relatively high temperatures and rainfall, promote the prolonged survival and development of the larval stages of *H. contortus* (Horak and Louw 1977; Mphahlele 2020).

The development of AHR poses a significant challenge to livestock health and productivity in resource-limited rural areas of South Africa (Mphahlele et al 2021). In this study, GINs, predominantly *Haemonchus contortus* were found to have developed resistance to the major anthelmintic classes including BZ, IDM, and ML. This finding is consistent with Mphahlele et al. (2021), who reported that *H. contortus* exhibited resistance to BZ, IMD, and ML, in communal farms of the Limpopo Province of SA. Tsotetsi et al. (2013) also detected anthelmintic resistance against three major anthelmintic drug classes on all five small ruminant farms surveyed in the Gauteng Province. Beyond SA, BZ resistance in GINs of small ruminants has recently been reported in Ethiopia (Maurizio et al. 2024) and Mozambique (Guinda et al. 2025). Highly ivermectin-resistant *H. contortus* populations have also been identified in sheep and goats in South Darfur, Sudan (Mohammedsalih et al. 2024).

This study found that on all selected eight sheep farms, *H. contortus* was resistant to at least two different anthelmintic drug classes. Similarly, more than two decades ago, Van Wyk et al. (1999) reported anthelmintic resistance in *Haemonchus* spp. on all 52 sheep farms surveyed in the Provinces of KwaZulu-Natal and Mpumalanga of SA. More recently, Mphahlele et al. (2021) found that 90% of sheep farms in the Limpopo Province had nematode strains resistant to at least one class of anthelmintics. In our study, Prodose® Orange (a co-formulation of a BZ + SLD) was found to be ineffective in controlling nematodes. This finding corroborates those of Van der Merwe (2021), who reported resistance to Prodose® Orange in *H. contortus* infecting Dohne Merino lambs at the Paradys experimental farm in Bloemfontein, Free-State Province, SA. Further afield, Claerebout et al. (2020) reported *H. contortus* resistance to an anthelmintic drug combination of mebendazole (BZ) and closantel (SLD) on sheep farms in Belgium. Lambertz et al. (2019) documented *H. contortus* resistance to a combination of oxfendazole (BZ) and closantel (SLD) in sheep in South Tyrol, Italy. The anthelmintic co-formulation of albendazole (BZ) and closantel (SLD) has been used to control GINs in sheep across various countries, including SA (Van der Merwe 2021), Australia (Khan et al. 1999), New Zealand (Miller and Leathwick 2012), Italy (Lambertz et al. 2019), and Mexico (Heredia et al. 2016). The use of dual combination is postulated to aid suppress the evolution of resistance by exerting multiple selection pressures on parasites (Whitley et al. 2022). Benzimidazole disrupts microtubules and impairs nutrient absorption, while SLD interferes with energy production and helminth movement (Furgasa et al. 2018). This synergistic action enhances the efficacy of BZ-SLD combinations against nematodes in ruminants (Dyary 2016). However, the effectiveness of this combination may be compromised by genetic mutations affecting the drug target site and alterations in membrane transporters that expel foreign substances, including anthelmintic drugs, from the cells (Mukherjee et al. 2023). Furthermore, this study found that Prodose® Red (IMD) was no longer effective in controlling of GINs in sheep on communal farms within the KSD local municipality. Similarly, Tsotetsi et al. (2012), reported resistance to levamisole (IMD) in GINs on small-scale sheep farms in Gauteng Province, SA. More recently, Mphahlele et al. (2021) detected levamisole resistance in GIN-infected sheep across four districts in Limpopo Province. Resistance to levamisole has also been documented on commercial sheep farms in Costa Rica (Castro-Arnaez et al. 2021). However, Aboelhadid et al. (2021) found that levamisole remained effective against *H. contortus* in Egyptian sheep. Imidazothiazoles act as allosteric modulators of glutamate-gated chloride channels in helminths. However, resistance can develop through alterations in nicotine acetylcholinesterase receptor function, preventing effective drug binding and resulting in reduced therapeutic response (Demessie et al. 2016).

The presence of multidrug resistance (MDR) observed in this study is consistent with the findings of Van Wyk and Bath (2002), who reported resistance to BZ, levamisole, and ML in small ruminants in SA. Similarly, Ahmed et al. (2014) documented MDR to ivermectin and a combination of abamectin and praziquantel in sheep at the livestock section of the University of KwaZulu-Natal, SA. Additionally, MDR to ivermectin, moxidectin, benzimidazole, and levamisole has also been reported in sheep populations in Kenya and Tanzania (Kamau et al. 2005), while Canto et al. (2017) observed MDR to combinations of closantel and fenbendazole, as well as closantel and albendazole on sheep farms in Mexico. This MDR phenomenon has been attributed to a genetic overlap in resistance mechanisms since each anthelmintic class targets different physiological pathways in nematodes (Mukherjee et al. 2023). Resistance to IDM and ML has been linked to mutations in P-glycoproteins and nicotine acetylcholinesterase receptors, which reduce the binding affinity of these drugs to their target sites in nematodes (Kalkal et al. 2020).

One limitation of this study is the absence of molecular diagnosis of the parasites present in the faecal samples prior to anthelmintic treatment. Although typical morphological keys were used to identify the GINs, the lack of molecular identification may have hindered a precise understanding of the parasite population diversity and anthelmintic resistance prior to treatment.

The anthelmintic resistance observed in this study may be attributed to several suboptimal helminth control practices identified on the selected sheep farms. Physical challenges associated with advanced age of the farmers can hinder the accurate administration of anthelmintics and the effective management of deworming schedules (Mthi and Nyangiwe 2018). In addition, inaccurate dosing and irregular application of anthelmintics, may further contribute to the failure of worm control measures (Mphahlele et al. 2019).

The availability and easy access to anthelmintic drugs at local veterinary drug shops in the KSD local municipality enable farmers to obtain these medications without a prescription. Moreover, these drugs are often administered without oversight from local veterinary authorities. The indiscriminate use of anthelmintics creates selection pressure on the parasites, ultimately leading to resistance (Charlier et al. 2022). Additionally, the failure to weigh sheep prior to drug administration, coupled with the use of syringes (5 mL/10 mL) instead of drench guns, results in inconsistent dosing. Inaccurate and incomplete parasite treatment may allow survival and the potential development of resistance (Nginyi et al. 2018). Furthermore, the lack of a drenching and shifting approach, where animals are continuously allowed to graze the same pasture, contributes to repeated exposure to the same parasite population. This ongoing exposure increases the likelihood of resistance development to anthelmintic drugs (Waghorn et al. 2009).

## Conclusion and recommendation

The findings from this study emphasise that anthelmintic resistance is a critical issue within the sheep farming sector in the communal areas of the KSD local municipality in SA. As a result, it is crucial to encourage sheep farmers to routinely monitor for anthelmintic resistance and receive training on the risk factors that contribute to its development. Additionally, the government should regulate the access to anthelmintics in local veterinary drug shops, ensuring they are only available with a prescription from a qualified veterinary professional. Local veterinary authorities should also provide supervision and guidance on the proper administration of anthelmintics. Future research should investigate the potential use of ethnoveterinary plant extracts for controlling helminths, reducing the sector’s dependence on chemical anthelmintics.

## Supplementary Information

Below is the link to the electronic supplementary material.

ESM 1 (DOCX 471 KB)

ESM 2 (DOCX 2.53 MB)

ESM 3 (DOCX 451 KB)

## Data Availability

No datasets were generated or analysed during the current study.
