# Supplementary material for: Anthelmintic resistance in gastrointestinal nematodes on communally reared sheep farms of the King Sabata Dalindyebo Municipality, South Africa
Source: Parasitol Res. 2025 Aug 5;124(8):86. doi: 10.1007/s00436-025-08532-x (PMC12325497; doi:10.1007/s00436-025-08532-x)
Supplement: Supplementary file 1 — (DOCX 471 KB) [file 436_2025_8532_MOESM1_ESM.docx]

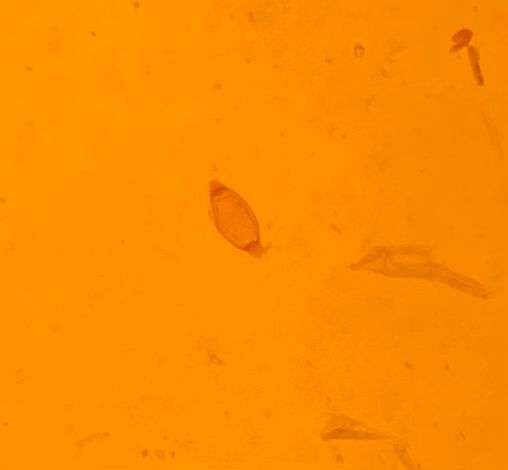

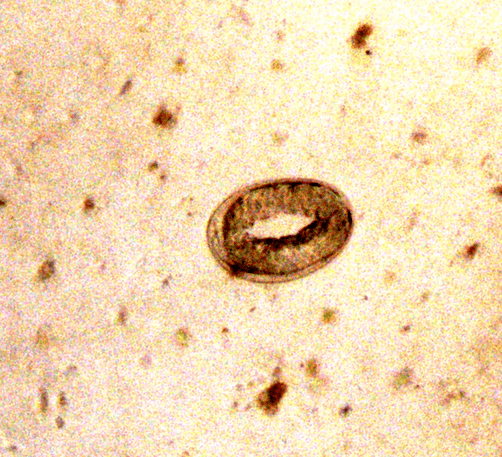

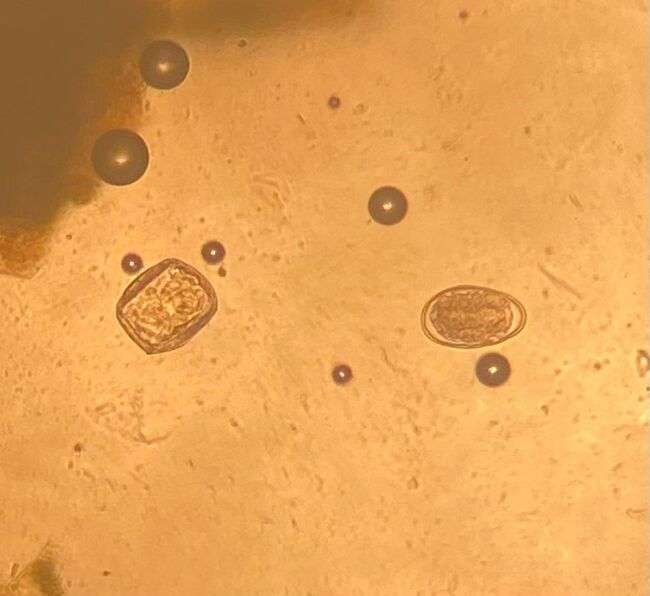

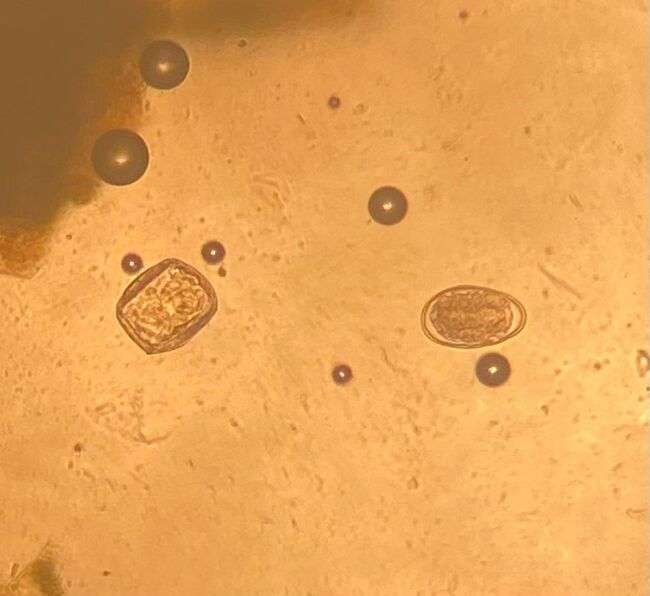


**D**:*Moniezia* spp egg

**A** :*Strongyloides* spp egg

**B** :Larvated *Strongyloides* spp egg

**E** :*Trichuris* spp egg


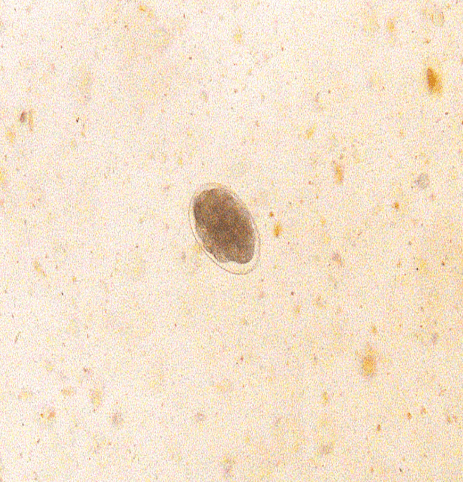


**F**: *Haemonchus* spp egg

**Supplementary Figure S1**: Micrographs of helminth eggs identified from faecal samples from lambs reared on communal farms of the KSD local municipality
