# Supplementary material for: Anthelmintic resistance in gastrointestinal nematodes on communally reared sheep farms of the King Sabata Dalindyebo Municipality, South Africa
Source: Parasitol Res. 2025 Aug 5;124(8):86. doi: 10.1007/s00436-025-08532-x (PMC12325497; doi:10.1007/s00436-025-08532-x)

**
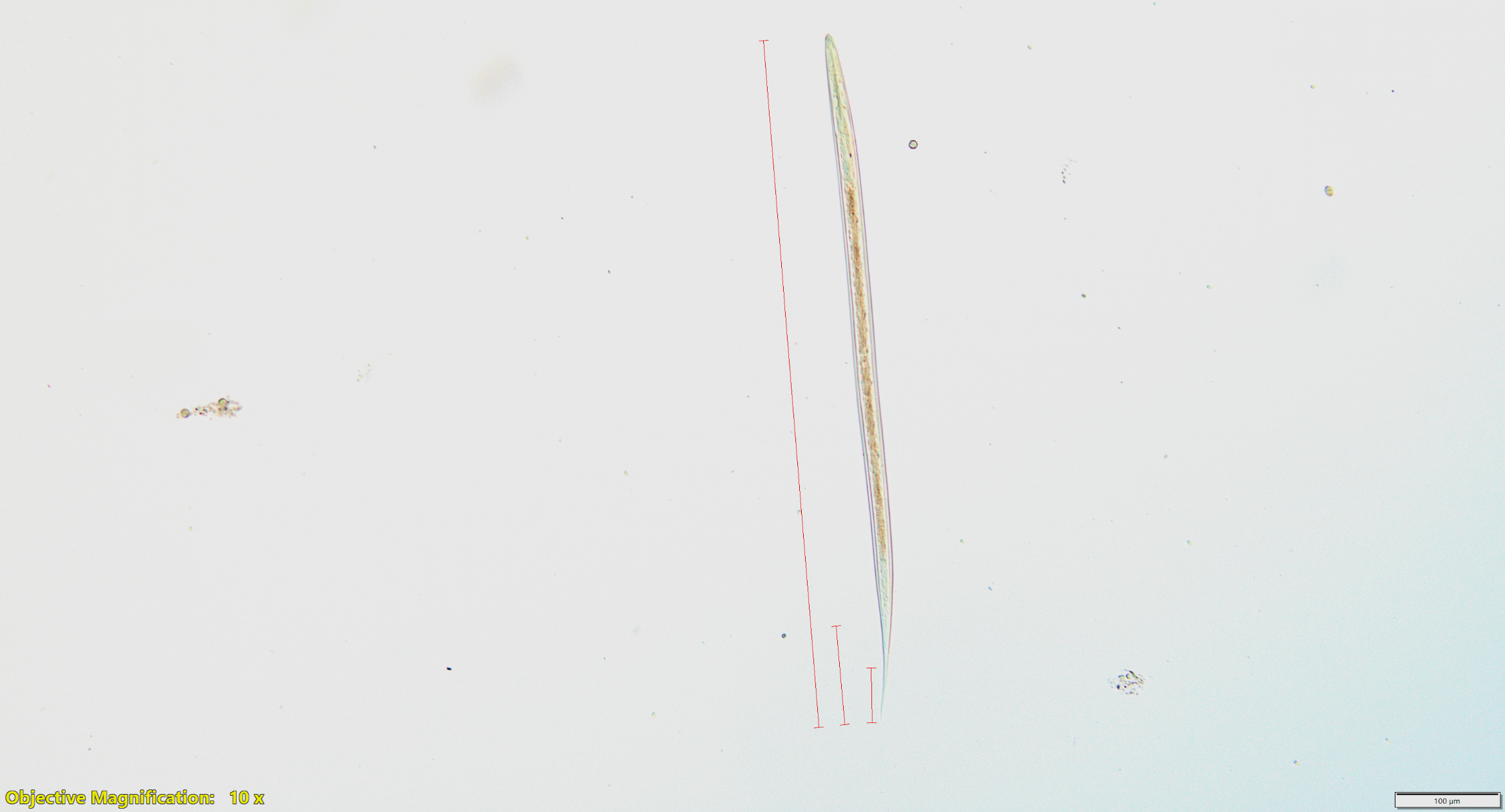
Supplementary Figure S2:** Third stage larva of *Haemonchus contortus* , depicting (a) tip of larva tail, (b) filament (54 µm), (c) sheath tail (97 µm), and (d) total length (679 µm).

c

d

b

a


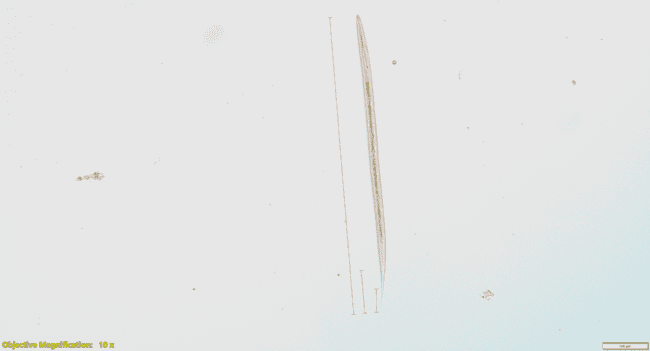

Supplement: Supplementary file 2 — (DOCX 2.53 MB) [file 436_2025_8532_MOESM2_ESM.docx]
