# Supplementary material for: Anthelmintic resistance in gastrointestinal nematodes on communally reared sheep farms of the King Sabata Dalindyebo Municipality, South Africa
Source: Parasitol Res. 2025 Aug 5;124(8):86. doi: 10.1007/s00436-025-08532-x (PMC12325497; doi:10.1007/s00436-025-08532-x)
Supplement: Supplementary file 3 — (DOCX 451 KB) [file 436_2025_8532_MOESM3_ESM.docx]

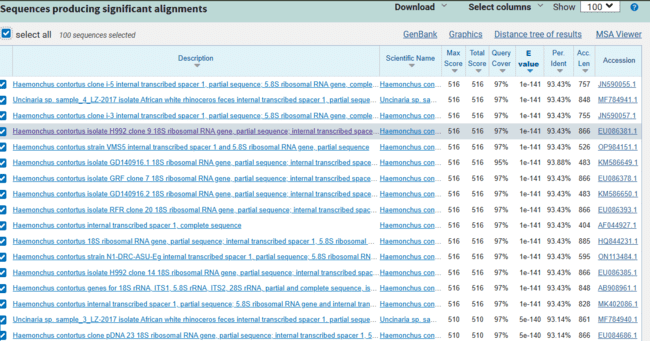


**Mgqumo Farm**


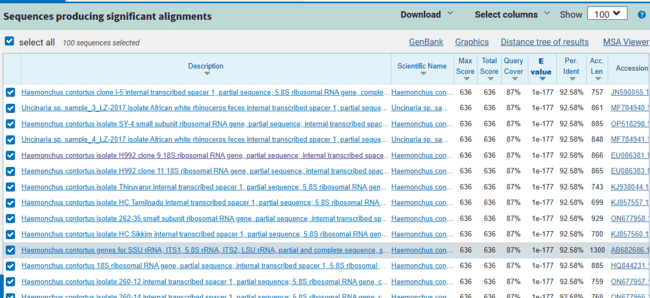


**Kwa-Ndamse Farm**

**Supplementary Figure S3:** BLASTn results showing the alignment of the *Haemonchus contortus* of this study and a corresponding sequence.


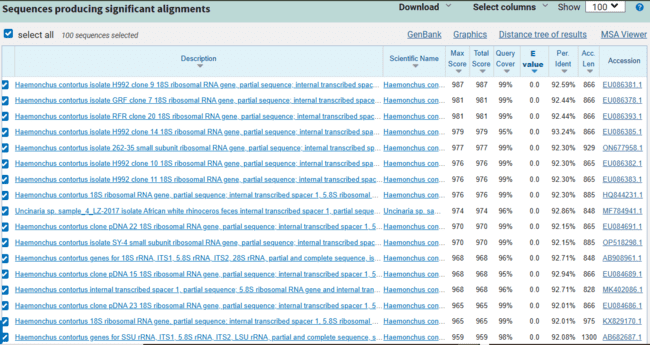


**Kwa-Xhakana Farm**
